# Diversity of BRAF mutations in non-small cell lung cancer and implications on treatment

**DOI:** 10.1038/s41698-025-01089-z

**Published:** 2025-10-28

**Authors:** Kevin Lu, John Paul Shen, Fernando J. Lopez-Diaz, Alessandro Leal, Isa Mambetsariev, Kaushal Parikh, Antonious Hazim, Brian D. Woodward, Abhinav Madduri, Faisal Khurshid, Jeremy Fricke, Vamsidhar Velcheti, Jonathan W. Riess, Aaron S. Mansfield, Ravi Salgia, Hatim Husain

**Affiliations:** 1 UC San Diego, La Jolla, CA USA; 2 MD Anderson Cancer Center, Houston, TX USA; 3 CEPIMP Genomics, Córdoba, AR USA; 4 NYU Langone, New York, NY USA; 5 City of Hope, Duarte, CA USA; 6 Mayo Clinic, Rochester, MN USA; 7 UC Davis, Sacramento, CA USA

**Keywords:** Non-small-cell lung cancer, Molecular medicine

## Abstract

The optimal treatment sequence in non-small cell lung cancer harboring class I *BRAF* mutations and atypical *BRAF* variants remains unclear. To better characterize therapeutic strategy, we retrospectively evaluated a multi-institutional cohort of *BRAF*-mutant NSCLC patients (*n* = 97) and an independent clinico-genomic database (*n* = 342), performed structural modeling, and conducted chemical screens of *BRAF*-mutant cell lines. Patients with class I *BRAF* mutation treated with BRAF–MEK inhibitors at any line of therapy had significantly greater median overall survival compared to those who did not receive BRAF–MEK inhibitors (40 vs 10 months, Log-rank *p* = 0.043). There, however, was no significant survival difference between patients treated with immune checkpoint inhibitors versus those not treated. Tumors with class II or III *BRAF* variants were significantly more likely to harbor concurrent MAPK pathway alterations relative to class I (Chi-Square *p* < 10^−4^). Cell line studies identified genetic dependency on *BRAF* in class II cell lines without sensitivity to BRAF inhibitors, and dependency on *EGFR* in class III cell lines.

## Introduction

Somatic mutations affecting the oncogene *BRAF* in lung cancer were first described in 2002 along with the initial observation of their oncogenic role in other solid tumors^1,2^. In a retrospective series of 1046 patients with non-small cell lung cancer (NSCLC), *BRAF* mutations were detected in 4.9% of adenocarcinoma and 0.3% of squamous cell carcinoma samples^3^. The well-known oncogenic variant V600E is most prevalent, accounting for approximately 40% of all *BRAF* mutations and 2% of all patients with NSCLC, followed by G469 and G466 which comprises 22% and 11% of *BRAF* mutations, respectively^4–7^. Targeted therapies have undergone regulatory approval for class I *BRAF*-mutated NSCLC and are under active investigation for class II and III^8^.

Combination anti-BRAF and anti-MEK therapy has demonstrated activity in *BRAF* V600E-mutant NSCLC. In 2017, dabrafenib and trametinib became the first combination targeted therapy FDA-approved for patients with advanced *BRAF* V600E-mutant NSCLC^9^. This approval is based on the phase II study BRF113928 (NCT01336634), which demonstrated a 63% overall response rate (ORR) and progression-free survival of 9.7 months (95% CI: 6.9–19.6) in previously treated patients (*n* = 57)^10^. Similar results were seen in the treatment-naïve cohort (*n* = 36) with 64% ORR and PFS of 10.9 months (95% CI: 7.0–16.6)^11^. In an updated report, median overall survival was 17.3 and 18.2 months for the treatment-naïve and pretreated cohorts, respectively^12^. In late 2023, the FDA approved encorafenib with binimetinib for *BRAF* V600E-positive metastatic NSCLC based on the phase II PHAROS trial (NCT03915951). Among 59 treatment-naïve patients, ORR was 75% while median duration of response (DoR) was not estimable (NE) (95% CI: 23.1–NE). Among 39 previously treated patients, ORR was 46% with a median DoR of 16.7 months (95% CI: 7.4–NE)^13,14^. However, these studies did not have arms that compared against immune checkpoint inhibitors, and the optimal timing of integration of BRAF–MEK inhibitors amidst other therapies remains less well defined.

There is less clarity about the functional significance and therapeutic implications of non-*BRAF* V600E mutations. Yao et al. characterized *BRAF* mutations into three functional classes based on data generated in preclinical models: class I mutations are defined as constitutively active monomers, class II mutations are active as dimers, and class III mutations have reduced kinase activity or are “kinase-dead”^15^. Class I *BRAF* variants, which include V600E, V600K, and V600L, constitutively activate the BRAF kinase, resulting in phosphorylation and downstream activation of MEK independent of RAS. Class II *BRAF* mutations also have increased kinase activity and act in a RAS-independent fashion; however, they are believed to require dimerization. These differ from class III variants, which are RAS-dependent dimers that may be oncogenic by allosteric interactions^15^. Since these variants are RAS-dependent, they are associated with alternate mutations such as *NF1* and *RAS*, which can promote dysregulation of RAS. Currently, there are no FDA-approved targeted therapies for class II or class III *BRAF* mutations, and a molecularly based therapeutic strategy for the atypical *BRAF* variant classes remain unclear.

In the current study, we evaluate a multi-institutional cohort of lung cancer patients with all classes of *BRAF* mutation receiving chemotherapy, immunotherapy, or targeted therapy to evaluate treatment efficacy for each class of *BRAF* mutation. To further evaluate class II and class III variants and provide insight into potential treatment opportunities for patients with these aberrations, we identified similarities and differences across various *BRAF* mutations using structural modeling, functional analyses with chemical and genetic perturbations, and co-alteration pathway assessment.

## Results

### Patient outcomes

To better understand the clinical impact of both atypical and *BRAF* V600E alterations, we performed a retrospective, multi-institutional study of advanced NSCLC with any *BRAF* alteration (*n* = 97 patients). The cohort had a slight female (54% vs. 46%) predominance which contrasts to a historical male predominance in oncogene-negative lung cancer^16,17^. The median age of the entire cohort was 66 years, ranging from 35 to 90 years. Former or current smokers were 62% of the cohort. In our cohort with class I–III variants, we observed a higher prevalence of current or prior smoking history in class II and III versus class I patients (9/12, 15/17, 24/46, respectively). The majority of patients were Caucasian (76%), followed by Asian (15%) and African–American or Black (5%) (Table 1a). For patients with first-line treatment data, 29 patients received cytotoxic chemotherapy, 16 received immunotherapy, and 11 received dabrafenib in combination with trametinib (including class 1 and 2 patients). The median overall survival of the entire cohort was 17.5 months.

**Table 1:** **A** Characteristics of the institutional *BRAF*-mutant NSCLC patient cohort; **B** Frequency of BRAF alteration within our clinical cohort and characterization of the mutations

A	
Characteristic	*N* (%)
Patients	97
Age at diagnosis (years)	
Median	66
Range	35-90
Age distribution	
<60 years	26 (27)
≥60 years	71 (73)
Gender	
Male	45 (46)
Female	52 (54)
Smoking history	
Former/current Smoker	60 (62)
Never smoker	37 (38)
Race	
White	74 (76)
Asian	15 (15)
Black/African–American	5 (5)
Non-White Hispanic	3 (3)
Lines of therapy	
0–2	69 (71)
3–5	24 (25)
>5	4 (4)
Mutation class/PD-L1	
Class 1	
≥50%	15 (52)
<49%	5 (17)
<1%	9 (31)
Class 2	
≥50%	2 (22)
<49%	4 (44)
<1%	3 (33)
Class 3	
≥50%	2 (13)
<49%	4 (25)
<1%	10 (63)

B						
BRAF mutation	*N*	Class (Yao et al.^15^)	Site	Kinase activity	RAS dependency	Dimer dependency
V600E/K/L	46	Class 1	Activation segment	High	No	No
K601E	6	Class 2	Activation segment	High	No	Yes
L597Q	1	Class 2	Activation segment	High	No	Yes
G469A	2	Class 2	P-Loop	Intermediate	No	Yes
G464V	1	Class 2	P-Loop	High	No	Yes
AGK-BRAF Fusion	2	Class 2	CR2 Region	High	No	Yes
N581S/I	9	Class 3	Catalytic loop	Low	Yes	Yes
G596R	3	Class 3	DFG motif	Low	Yes	Yes
G469E	1	Class 3	P-Loop	Low	Yes	Yes
D594H/N	3	Class 3	DFG motif	None	Yes	Yes
G466V	1	Class 3	P-Loop	Low	Yes	Yes

Unclassified BRAF alterations not represented include: D22N, E24E, P25S, E26D, A31G, S113I, P132S, K150K, R444W, G469*, E501K, L537L, I573N, H574R, L597R, K630*, D638E, R726S, I755V. Class activity is described by Yao et al.^15^.

**Fig. 1:**
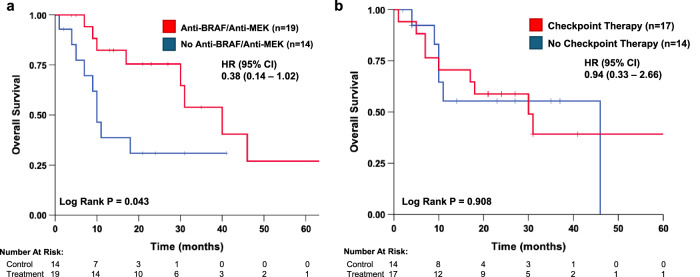
Survival analysis of patients with advanced lung cancer harboring class I BRAF mutations. **a** Survival analysis of patients with class I BRAF mutations who did and did not receive anti-BRAF and anti-MEK dual therapy. The estimated median OS for those receiving targeted therapy and those without was 40 months and 10 months, respectively. **b** Survival analysis of patients with class I BRAF mutations who did and did not receive immune checkpoint therapy. The estimated median OS for those receiving immunotherapy and those without was 30 months and 46 months, respectively.

Class I *BRAF* mutations were detected in 46 of 97 patients (47%), class II mutations in 12 patients (12%), and class III in 17 patients (18%). The most frequent class II and III mutations were K601E (*n* = 6) and N581S/I (*n* = 9), respectively. In addition, we identified two patients with AGK-BRAF fusion mutations and three with intron rearrangements. Three different mutations in codon 469 were detected, including the class II G469A, class III G469E and non-classified G469* (Table 1b). For BRAF targeted therapy across all lines of therapy, 21 patients with *BRAF* V600E received dabrafenib and trametinib, while one patient received vemurafenib and cobimetinib. BRAF-MEK dual targeted therapy was first-line for nine patients, second-line for six patients, and third-line or greater for seven patients (Supplemental Fig. 1). Patients with class I *BRAF* mutation who received anti-BRAF and anti-MEK therapy at any line (*n* = 19) had significantly superior overall survival compared to class I *BRAF* mutation patients who did not receive dual targeted therapy (median OS 40.0 vs. 10.0 months; Log-rank *p* = 0.043, Fig. 1a). In contrast, there was not a significant difference in overall survival comparing class I *BRAF* patients treated with immune checkpoint inhibitors to class I *BRAF* patients who did not receive immunotherapy (median OS 30.0 vs. 46.0 months; Log-rank *p* = 0.908, Fig. 1b). Immunotherapy median time to treatment discontinuation appeared similar across all classes: class I = 5.0 months (*n* = 16), class II = 4.0 months (*n* = 3), and class III = 3.0 months (*n* = 7).

### Co-mutation and molecular analysis

**Fig. 2:**
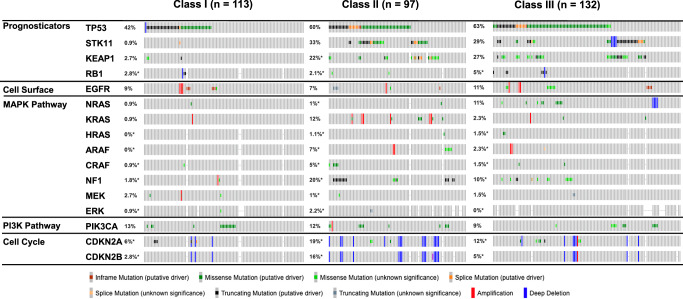
A comparison of co-occurring genetic alterations between BRAF classes from NSCLC samples derived via cBioPortal. There was a significant difference in co-alterations between BRAF classes determined by Pearson chi-square analysis (*X*^2^ (2, *N* = 342) = 30.68, *p* < 0.001). Further post hoc testing showed that there were significantly fewer patients with MAPK pathway mutations in the class 1 cohort (*p* < 10^−5^) and significantly more in class 2 (*p* < 10^−4^). Percentage of patients with alterations in MAPK pathway genes surveyed: Class I = 7%, Class II = 39%, Class III = 26%.

To better understand cancer dependencies, we sought to characterize the co-occurring genomic alterations of class I, class II, and class III *BRAF* variants in NSCLC using data available in the cBioPortal clinico-genomic database distinct from our institutional cohort (Class I *n* = 113, Class II *n* = 97, Class III *n* = 132)^18,19^. *TP53* was the most common co-altered gene observed. Tumors with class I *BRAF* mutations rarely have other genetic alterations in the MAPK pathway (8/113, 7.1%, Chi Square *p* < 10^−5^), suggesting that in the setting of the strong constitutive activation of *BRAF* there is limited dependency on additional MAPK activating mutations (Fig. 2). In contrast, tumors with class II and III *BRAF* mutations had frequent alterations in other MAPK genes, significantly more in class II when compared to class I (Class II 38/97 = 39.2%, Class III 34/132 = 25.8%; Class II Chi Square *p* < 10^−4^ and Class III Chi Square *p* = 0.73). A total of 33 RAS alterations (15 *NRAS*, 15 *KRAS*, 3 *HRAS*) were seen in class II or III tumors; however, two were seen in class I *BRAF*-mutated tumors. Truncating mutations in *NF1*, a known negative regulator of MAPK signaling, and activating mutations in *CRAF* were more common in tumors with class II *BRAF* mutations^20^. Five *CRAF* mutations (4 S259F, 1 G24V) were observed in class II, while two in class III (P261L & S259T). Activating mutations in *NRAS* were particularly frequent in tumors with class III *BRAF* mutation (10 somatic mutations and 4 deletions). Apart from the MAPK pathway, we also observed that *STK11* and *KEAP1*, which are also often co-mutated in *KRAS*-mutant NSCLC, are more frequently co-mutated with class II or III *BRAF* mutations compared to class I variants^21^. *STK11* alterations co-occurred in 33% (32/97) class II *BRAF*-mutated patients and 29% (38/132) class III *BRAF-*mutated patients. *KEAP1* alterations were observed in 22% (21/95) of class II and 27% (36/132) of class III patients. *STK11* and *KEAP1* mutations were nearly absent in the class I cohort. To better understand the potential applications of immunotherapy in *BRAF*-mutant NSCLC, we analyzed the tumor mutation burden and the fraction genome altered between all three *BRAF* mutation classes using data from cBioPortal. *BRAF* class I mutant tumors had significant lower median tumor mutation burden (4.8 mut/Mb vs. 8.65 mut/Mb Class II Wilcoxon *p* = 1.77e^−9^, 10.8 mut/Mb Class III Wilcoxon *p* < 10^−10^) and fraction genome altered when compared to class II and III (0.1 vs. 0.15 Class II Wilcoxon *p* = 0.021, 0.19 Class III Wilcoxon *p* = 9.31e^−4^ Supplemental Fig. 2). In analyzing our dataset to compare the prevalence of PD-L1 expression between classes, 54% of class I mutant patients had a PD-L1 greater than or equal to 50%. A smaller percentage of class II and class III patients had a greater than or equal to 50% PD-L1 expression (22% and 13% respectively, Table 1a). Across PD-L1 status, the time to discontinuation of anti-PD1 immunotherapy ranged from 1 to 38 months for the PD-L1 high subgroup >50%, 1–7 months for patients with PD-L1 1–49%, and 1–13 months for PD-L1 negative patients. For the subgroup of BRAF-altered patients with a prior smoking history, the time to discontinuation of anti-PD1 immunotherapy was 1–38 months. The time to anti-PD1 immunotherapy discontinuation was 1–20 months for never-smoking patients.

### Structural modeling

**Fig. 3:**
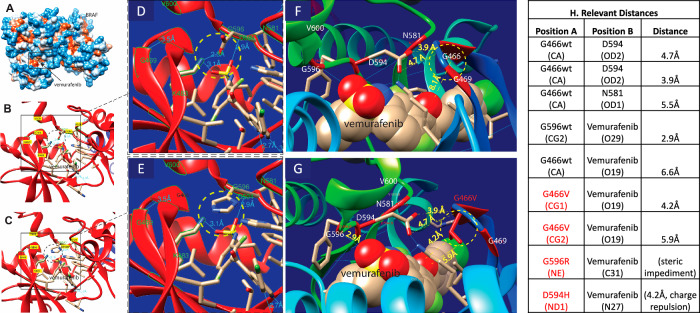
In silico modeling of BRAF 3D class III hotspot domain in the kinase domain interaction with vemurafenib (PDB:4RZV). **a** Space-filling hydrophobicity surfaces model of BRAF and vemurafenib. **b** View of the domain encompassing all class III hotspots. The BRAF-vemurafenib interaction with the D594wt BRAF protein is shown. **c** View of in-silico modeling of the 3D class III hotspot domain. The most stable rotamer substitution for class III D594H BRAF mutant, causing both an intrusion into the compound pocket and a rupture of H-bonds to vemurafenib, is shown on the model as an example. **d** Zoom of figure (**b**). **e** Zoom of figure (**c**). All class III hotspot sites, plus relevant positions including V600, K483, and G596, are highlighted (green). Hydrogen bonds between vemurafenib-BRAF or intra-chain interactions on the binding pocket are shown in blue. **f** vemurafenib (spherical model) pocket occupation on wt 3D class 3 hotspots (red) domain. **g** The circled region shows the aliphatic chain as a result of the G466V substitution. Wild-type V600 is farther apart from vemurafenib than all class III hotspots. **h** Table showing relevant atomic distances in Angstrom (Å).

Three-dimensional space-filling hydrophobicity surface modeling of class III (G466V, G596R, D594H) *BRAF* mutations was performed using Chimera software^22,23^. Simulation of the most stable rotamer substitution for class III mutation *BRAF* D594H revealed that the size of the ATP binding pocket changed depending on the specific aberration identified. For example, we noted that vemurafenib fit in a hydrophobic pocket (Fig. 3a) involving a short (2.3 Å) hydrogen bond between an oxygen atom at G594 and Lys 483 atom, and two Hydrogen bonds between its oxygen 29 and 30 on vemurafenib Lys 483 Hydrogen (2.9 Å) and with another hydrogen Gly 596 (3.1 Å), respectively (Fig. 3b, d). Interestingly, we observed that V600 is at a much greater distance. In the D594H mutant, however, the H-bond between oxygen at D594 and vemurafenib is lost (Fig. 3c, e), which may explain why vemurafenib does not appear to inhibit class III mutant BRAF protein.

### In vitro analyses across BRAF Class II and III variants in NSCLC

**Fig. 4:**
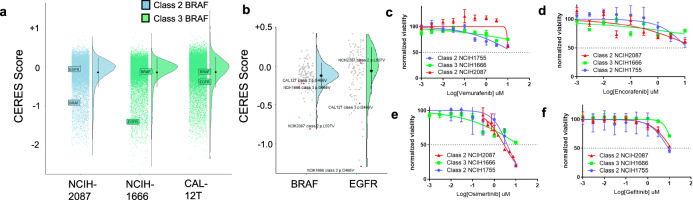
Pathway dependencies and pharmacologic inhibition of BRAF class II and III NSCLC cell lines in vitro. Mutations for each cell line are defined as follows: Class II BRAF L597V (NCIH-2087), Class III BRAF G466V (NCIH-1666), Class III BRAF G466V (CAL-12T). The CERES score is a calculated metric quantifying cell line-gene dependency. A score of zero suggests a lack of dependency, while negativity indicates stronger dependency. **a** A relationship analysis between gene knockout and mutant *BRAF* NSCLC cell lines. **b** A relationship analysis of gene dependency and cell line. **c**–**f** Selected pharmacologic inhibition of *BRAF* class II and III NSCLC cell lines in vitro. Drug concentration is displayed on a logarithmic scale. Molecular inhibitors used from panels (**a**–**d**), respectively: vemurafenib, encorafenib, osimertinib, and gefitinib.

We leveraged functional genomic data from the DepMap software to better understand oncogenic addiction to atypical *BRAF* mutations. It is well established that NSCLC tumors with *BRAF* V600E mutation display oncogene addiction to *BRAF*; however, there are no NSCLC *BRAF* V600E cell lines in the DepMap^24^. CRISPR knockout data were available for a class II cell line (NCI-H2087) and two class III cell lines (NCI-H1666 and CAL-12T). In the class II mutant cell line NCI-H2087, *BRAF* knockout resulted in significant loss-of-fitness (Fig. 4a). Of the 161 NSCLC cell lines in the DepMap, *BRAF* knockout had the greatest effect on NCI-H2087 (Fig. 4b). These data suggest that class II mutations induce oncogene addiction to *BRAF*. In contrast, the cell lines with class III *BRAF* mutations did not display oncogene addiction to *BRAF*; knockout of *BRAF* in these cell lines was no more detrimental than *BRAF* knockout in other NSCLC cell lines (Fig. 4b). Interestingly, the NCI-H1666 cell line was quite sensitive to *EGFR* knockout. These results suggest that some class III mutant NSCLC tumors may be sensitive to EGFR inhibition, similar to reports in colorectal cancer^25^.

In order to evaluate the effect of pharmacologic inhibition of the MAPK pathway in cell lines with class II and III *BRAF* mutations, we performed cell viability assays against a panel of small-molecule kinase inhibitors (Fig. 4c–f). Neither vemurafenib nor encorafenib, BRAF inhibitors designed specifically for *BRAF* V600E mutations, showed meaningful activity against cell lines with class II or III *BRAF* mutations. The first-generation EGFR inhibitor gefitinib showed activity only at high doses, while the covalent EGFR inhibitor osimertinib showed modest activity against both cell lines with class II *BRAF* mutation.

## Discussion

As the landscape of lung cancer therapies continues to evolve, it has become clear that further work to understand the appropriate personalization of therapy is critical in this subset of disease. The categorization of *BRAF* alterations based on the functional characteristics described by Yao et al. can be extended with additional dependency analyses, functional analyses, and emphases placed on co-associated genes. We believe that the work here provides a model to re-evaluate the previous classifications of Yao et al. to include new ways to categorize some of the unclassified atypical *BRAF* aberrations and identify therapeutic implications.

We observed that the longest survival for *BRAF* V600E-mutant patients was seen in those who were treated with anti-BRAF and anti-MEK directed therapy, irrespective of receiving checkpoint immunotherapy. This finding supports that anti-BRAF directed therapy may contribute to longer OS for patients with class I alterations and is most reliably ensured by front-line therapy use. An update on the PHAROS trial was presented at ESMO24 and was notable for an observed median OS that was not reached (95%: CI 31.3 months–NE) in the treatment naïve cohort^26^. While anti-BRAF and anti-MEK therapy have not been compared to immunotherapy in a randomized clinical trial setting, guidelines have recommended first-line targeted therapy administration^27^. Providing this therapy in the front line ensures the availability of this combination for patients who may not receive additional lines of therapy. Data across other studies have shown that approximately 50% of patients may not receive subsequent therapy after first-line treatment^8^. The majority of class I patients on anti-BRAF and anti-MEK therapy (10/15) were still alive at last follow-up (Supplemental Fig. 1). Retrospective studies have demonstrated mixed efficacy of immunotherapy for class I BRAF-mutated patients. In the IMMUNOTARGET registry study of immunotherapy in oncogene addicted NSCLC, the *BRAF* altered subgroup (*n* = 43), which included V600E and non-V600E, had a median PFS of 2.5 months^28^. However, in another retrospective analysis of immunotherapy in advanced driver mutant NSCLC, the median PFS of the BRAF V600E group was 9.8 months^29^. In our study, we did not observe an overall survival advantage associated with immunotherapy for class I patients.

Structural modeling was used to determine the binding distances across the protein to BRAF inhibitors and identified steric impediments and charge repulsions that predicted a lack of response to selective BRAF inhibitors (Fig. 3). We speculate that the steric impediment for vemurafenib, in combination with other factors, may be associated with insensitivity to BRAF inhibition. This is further corroborated by the lack of class II and class III cell line sensitivity to BRAF inhibitors observed (Fig. 4c, d). Alternative strategies may be required for atypical *BRAF* mutations. Further silica modeling is required to corroborate these findings.

In our cohort, class II and III mutants were commonly associated with co-occurring MAPK-activating aberrations. In contrast, few co-associated MAPK alterations were observed in class I mutants, and we speculate that in the setting of the strong constitutive activation of *BRAF*, there may be more limited reliance on additional MAPK activating mutations. A prior pan-tumor study has observed a similar statistically significant association of MAPK alterations in non-V600E mutant tumors^30^. We observed a significantly elevated TMB and fraction of genome altered in the class II and III patient subset compared to that observed in subjects with class I variants. Pre-clinical data suggest that MAPK pathway activation resulting from BRAF activating alterations (including non-V600E) may be sensitive to targeting downstream signaling through MEK and ERK^15^. However, we did not observe much efficacy of pan-RAF, ERK, MEK, and pan-EGFR inhibitors in class II or class III cell lines as single agent therapy (Supplemental Fig. 3). Further analyses are needed to determine the clinical value of EGFR, ERK, MEK, and pan-RAF inhibition in class II and III patients, and combinatorial strategies.

Several novel BRAF-targeting agents in development have been positioned to address unmet needs in *BRAF*-mutant NSCLC, including atypical non-V600E *BRAF* variants. Combinatorial strategies, including pan-RAF and non-RAF inhibition, are under active investigation. Selected compounds in the developmental pipeline include: PLX8394 (BRAF dimerization inhibitor, Novellus/Plexxikon), VS-6766/RO5126766 (RAF/MEK inhibitor, Verastem), LY3214996 (ERK1/2 inhibitor, Eli Lilly), LXH254 (BRAF/CRAF inhibitor, Novartis), and lifirafenib/BGB-283 (RAF dimer inhibitor, BeiGene)^8,30–33^. Currently, there is an active phase 1/1b trial (NCT04913285) evaluating KIN-2787 (pan-RAF inhibitor, Kinnate) in *BRAF* and *NRAS* mutation-positive solid tumors^34^. Additionally, a phase 1b/2 trial (NCT04985604) is investigating tovorafenib (BRAF/CRAF inhibitor, DayOne) and pimasertib (MEK1/2 inhibitor, Merck KGaA) for solid tumors, including NSCLC harboring MAPK pathway mutations^35^.

In conclusion, *BRAF* variants have distinct functional and therapeutic implications. For patients with *BRAF* V600E mutations, survival in our cohort was longest when an anti-BRAF and anti-MEK therapy was included in treatment. Redefining *BRAF* classes based on structure and function analyses provides an opportunity to facilitate future directions for therapeutic advancement for this diverse oncogene in NSCLC.

## Methods

### Crystalline structure and cell line design

Modeling of the protein BRAF with specific mutations was performed using the software package UCSF Chimera^22,23^. Prior studies have utilized UCSF Chimera to model the electrostatic repulsion of mutant *BRAF*^36,37^. BRAF wild-type and mutant protein structures were modeled on the crystal structure of the BRAF kinase domain complexed with vemurafenib (PDB:4RZV)^38^. The Dependency Map (DepMap) database was used to evaluate functional dependencies of NSCLC cell lines harboring class II and class III mutants^24^. Unfortunately, at the time of analysis, there were no NSCLC cell lines with class I *BRAF* mutations within the DepMap repository. Cell lines selected to examine for in vitro testing included NCI-H2087, NCI-H1666, and NCI-1755. In vitro cell viability studies were conducted with class II and III cell lines, which were tested against pan-RAF, MEK, ERK, and EGFR inhibitors to determine functional dependence and therapeutic associations.

### Clinico-genomic database analysis

We evaluated clinical characteristics and co-alterations of each *BRAF* class on the publicly available cancer genomics database, cBio Cancer Genomics Portal (cBioPortal)^18,19^. We identified and built our query search from fourteen datasets within cBioPortal that included either non-small cell lung cancer or lung adenocarcinoma samples. The studies originated from TCGA, MSKCC, Broad, TRACERx, TSP, and OncoSG. We used the *BRAF* mutation classifications defined by Yao et al. to construct an analysis of co-occurring alterations (OncoPrint)^15^. The co-mutations we chose to represent in the co-alteration analysis were selected due to knowledge of the downstream pathway activity, mediators, alternate signaling pathways, and cell cycle regulators. We structured our analysis around the *RAS*, *RAF*, *PIK3CA*, and cell cycle families. In addition to identifying co-alterations, we analyzed tumor mutation burden (TMB) and the fraction of the genome altered by copy number variation (CNV) among the samples within the query search. TMB was reported and assessed based on multiple assays of different sequencing depth from studies, including TCGA, MSKCC, Broad, TRACERx, TSP, and OncoSG. Tumor samples were sequenced at varying clinical timepoints as outlined by the corresponding study above.

### Multi-institutional cohort review

We compared co-occurring alterations to those discovered in an independent database of *BRAF*-mutant patients from a multi-institution collaboration at the University of California, City of Hope, NYU and Mayo Clinic. We reviewed all patients with BRAF alterations at our institutions from 2009 to 2021 and have presented this cohort in a CONSORT diagram (Supplemental Fig. 4). In this cohort (*n* = 120), 23 patients had BRAF amplifications while 97 patients had BRAF mutations. We examined all of our 97 NSCLC patients who were found to have *BRAF* mutations during routine clinical care. Overall survival for all patients was defined from the date of diagnosis to the date of death or last follow-up. Only *BRAF* mutant patients who received treatment were included in the analysis. All patients had locally advanced or metastatic disease at the time of the study and a histology diagnosis of NSCLC. Since clinical outcome data were acquired by retrospective review, first-line treatment outcome was available for 82 of 97 patients. A direct comparison of first-line targeted therapy versus immunotherapy, then targeted therapy was not possible because most patients did not receive sequential use of targeted therapy and immunotherapy.

All tissue testing was performed by multi-gene, broad NGS assays at the time of diagnosis. Our study reflects a real-world cohort with testing performed based on institutional standards across clinically available NGS platforms. For a subset of patients, we applied a hybrid capture-based targeted enrichment approach that detects genomic alterations in 315 cancer driver genes, including base substitutions, short insertions/deletions, copy number alterations, as well as fusions via intron baiting for 31 genes (Foundation Medicine; Cambridge, MA, USA).

Clinical characteristics, including age at diagnosis, gender, ethnicity, smoking status, histology, sites of metastatic progression, and treatment history with response information, were provided by the treating physician (Table 1a). The study was approved by Institutional Review Boards at the City of Hope Cancer Center, University of California Davis, New York University, Mayo Clinic, and University of California San Diego.

## Supplementary information

BRAF Supplemental Figures

## Data Availability

All data supporting this study’s findings are available within the paper and its supplementary information files. Gene expression data will be uploaded to GEO.
