# Supplementary material for: Diversity of BRAF mutations in non-small cell lung cancer and implications on treatment
Source: NPJ Precis Oncol. 2025 Oct 28;9:335. doi: 10.1038/s41698-025-01089-z (PMC12568928; doi:10.1038/s41698-025-01089-z)
Supplement: Supplementary file 1 — BRAF Supplemental Figures [file 41698_2025_1089_MOESM1_ESM.pdf]

**Supplemental Figure 1: Time to Treatment Discontinuation of Systemic Therapy By BRAF Class**

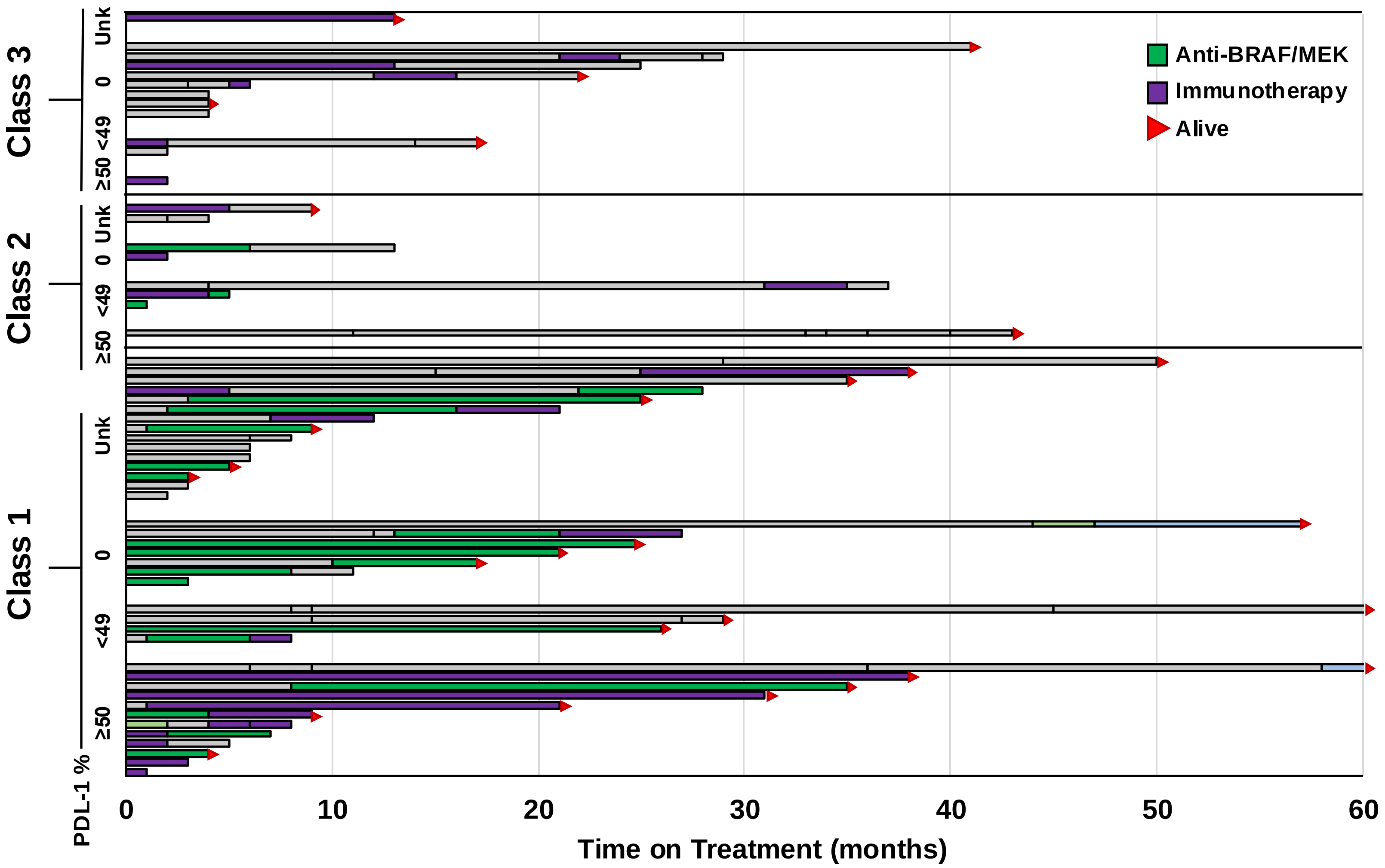


**Supplemental 1. Time to treatment discontinuation for each line of systemic therapy.** Patients are grouped based on BRAF class and PDL-1 status. Arrows depict patients who are still alive at study cutoff. Bars shaded in gray represent non-combinatory BRAF/MEK or immunotherapy treatments. Two patients had a cumulative time on therapy that surpassed 60 months with a dabrafenib and trametinib time to treatment discontinuation of 23 and 30 months not displayed on the plot.

## Supplemental Figure 2: Comparison of TMB and FGA Between BRAF Classes

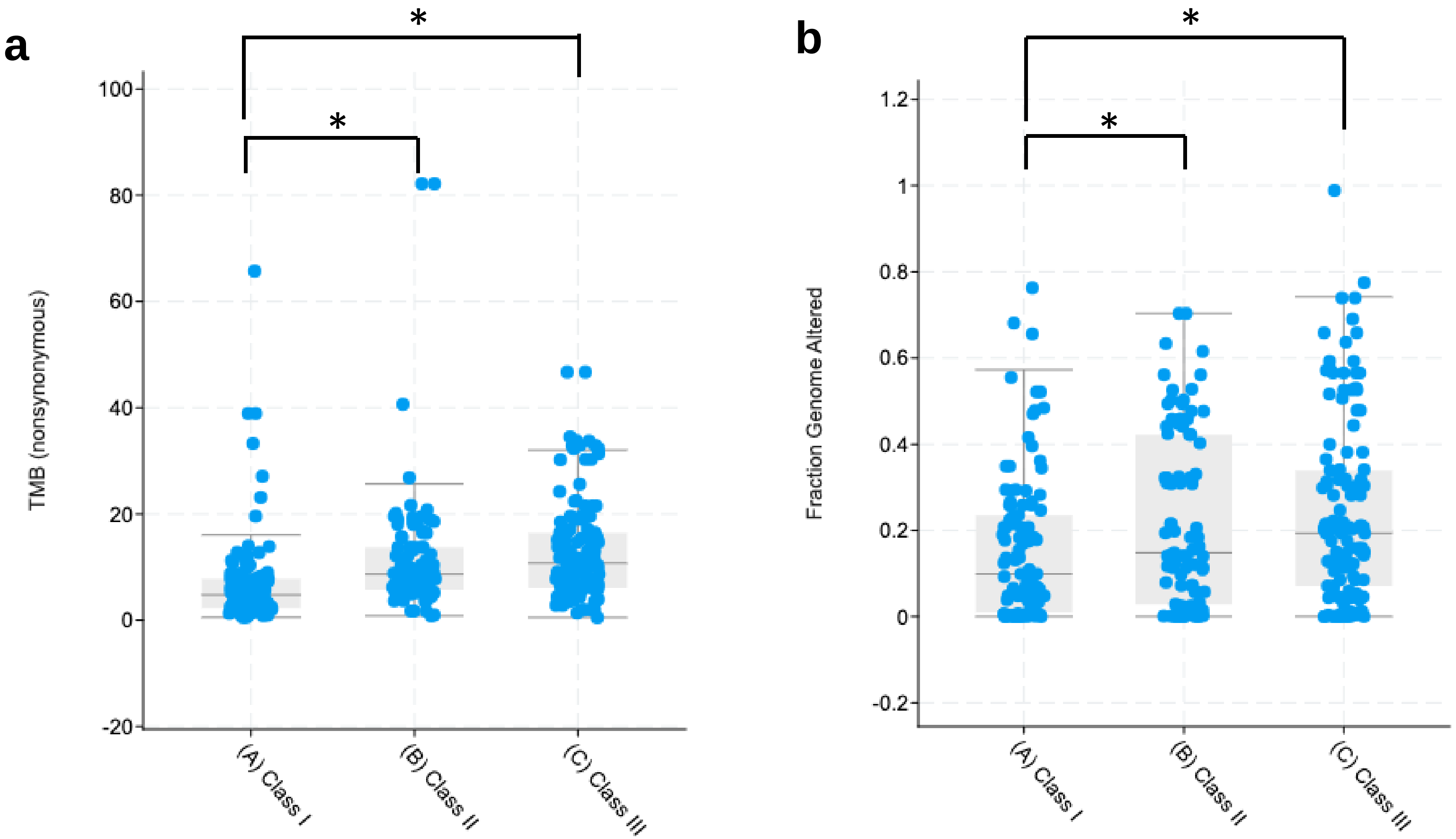


**Supplemental 2. a)** Tumor mutation burden (TMB) of *BRAF*-mutant NSCLC patient samples from cBioPortal (class I = 114, class II = 105, class III = 142). Significant differences per Wilcoxon testing were observed between class I and II ($p=1.77e^{-9}$) in addition to class I and class III ($p<10^{-10}$). **b)** Median fraction genome altered (FGA) of *BRAF*-mutant patient sample from cBioPortal (class I = 110, class II = 91, class III = 125). Significant differences per Wilcoxon testing were also observed between class I and II (p=0.021) in addition to class I and class III ($p=9.31e^{-4}$). Asterisk represents statistically significant difference between classes.

## Supplemental Figure 3: *In Vitro* Pharmacologic Screening of BRAF Class II and III NSCLC Cell Lines Against Molecular Inhibitors

| | EGFR | | EGFR | | EGFR | | BRAF V600E | | BRAF V600E | | pan-RAF | | pan-RAF | | ERK | | | | MEK | | pan-RAF + ERK | |
|---|---|---|---|---|---|---|---|---|---|---|---|---|---|---|---|---|---|---|---|---|---|---|
| | Afatinib | | Gefitinib | | Osimertinib | | Encorafenib (LGX818) | | Vemurafenib (PLX4032) | | LY3009120 | | PLX8394 | | LY3214996 | | | | Trametinib | | LY3009120 (fixed) + LY3214996 | |
| | IC50 | AUC | IC50 | AUC | IC50 | AUC | IC50 | AUC | IC50 | AUC | IC50 | AUC | IC50 | AUC | IC50 | IC50 (rep) | AUC | AUC (rep) | IC50 | AUC | IC50 | AUC |
| NCIH-1755 | 6.3 | 364 | 8.9 | 356 | 4.0 | 348 | >10 | 386 | >10 | 343 | >10 | 355 | >10 | 452 | >10 | | 407 | | >10 | 384 | >10 | 265 |
| NCIH-2087 | 1.2 | 299 | >10 | 371 | 2.8 | 90 | >10 | 352 | >10 | 468 | >10 | 281 | >10 | 313 | >10 | | 312 | | 0.6 | 232 | | |
| NCIH-1666 | 8.4 | 316 | >10 | 399 | >10 | 325 | >10 | 339 | >10 | 350 | >10 | 372 | >10 | 350 | >10 | >10 | 326 | 311 | >10 | 290 | >10 | 286 |

**Supplemental 3. A diverse multi-drug inhibition analysis of *BRAF* class II and III NSCLC cell lines *in vitro*.** Mutations for each cell line defined as following: Class II BRAF L597V (NCIH-2087), Class II BRAF G469A (NCIH-1755), Class III BRAF G466V (NCIH-1666).

**Supplemental Figure 4: CONSORT Diagram for Institutional Cohort Analysis**

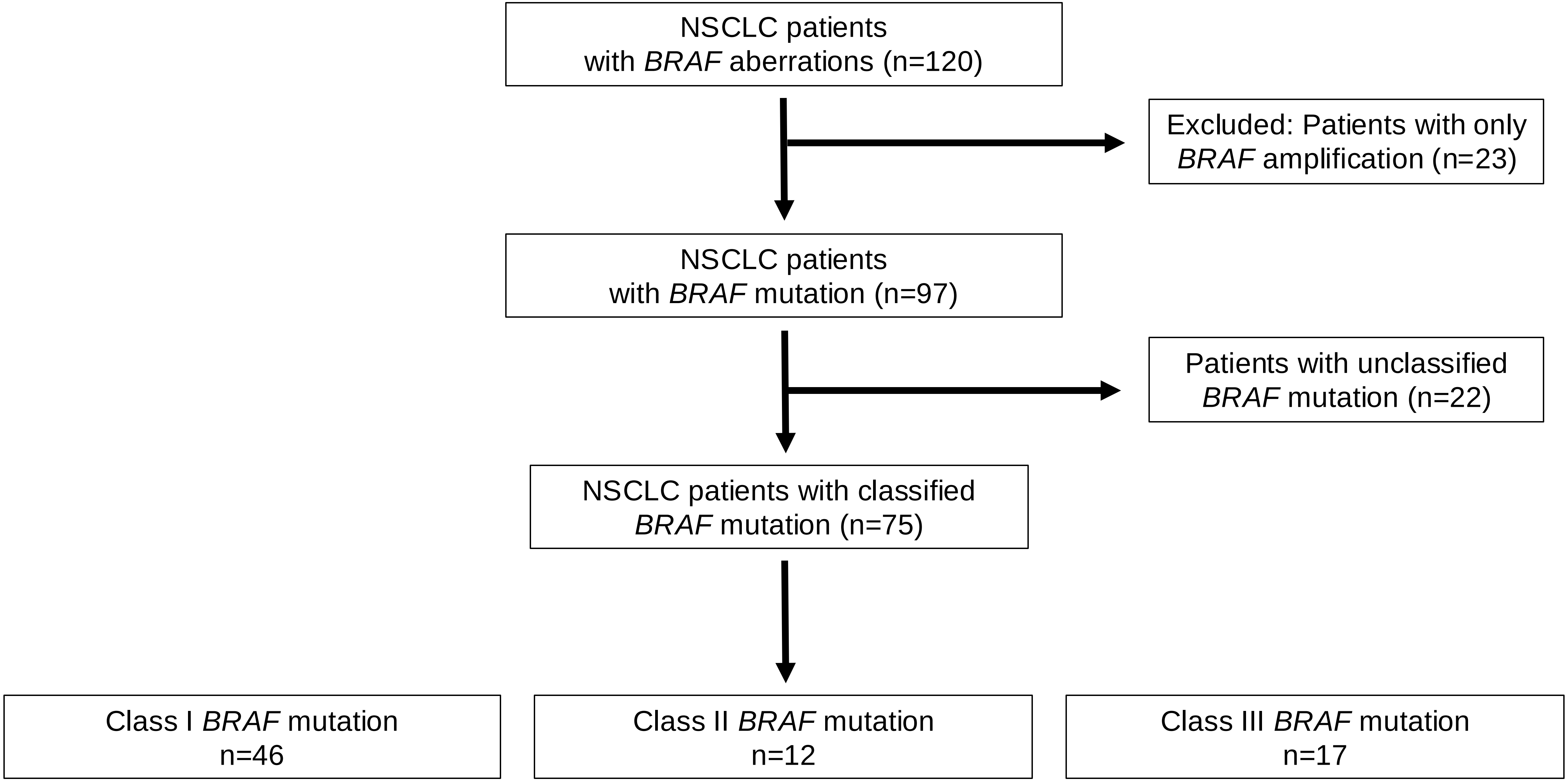


**Supplemental 4. Flow diagram of study population from multi-institutional sites.** Initial population selected for patients with NSCLC harboring *BRAF* aberrations and excluded patients with *BRAF* amplification as their only *BRAF* aberration.
